# Estimated Prevalence of US Physicians With Disabilities

**DOI:** 10.1001/jamanetworkopen.2021.1254

**Published:** 2021-03-12

**Authors:** Zakia Nouri, Michael J. Dill, Sarah S. Conrad, Christopher J. Moreland, Lisa M. Meeks

**Affiliations:** 1 Workforce Studies, Association of American Medical Colleges, Washington, DC; 2 Department of Internal Medicine, Dell Medical School, The University of Texas at Austin; 3 Center for a Diverse Healthcare Workforce, University of California, Davis, School of Medicine, Sacramento; 4 Department of Family Medicine, The University of Michigan Medical School, Ann Arbor

## Abstract

This survey study uses data from the Association of American Medical Colleges National Sample Survey of Physicians to assess the prevalence and characteristics of US physicians with disabilities among a survey of 6000 practicing physicians.

## Introduction

Disability is an increasingly important topic in medical education,^1^ and recent association guidance has recognized it as an essential component of diversity.^2,3,4,5^ Although information about the prevalence of disabilities among medical students is well established,^4,5^ there are no parallel data for physicians. To our knowledge, our survey study represents the first systematic report of the prevalence and characteristics of practicing physicians with disabilities using data from the Association of American Medical Colleges 2019 National Sample Survey of Physicians.

## Methods

The National Sample Survey of Physicians collected data from a sample of 6000 practicing physician members of a health care professional panel (eMethods in the Supplement). A total of 86 951 qualified physicians were invited to participate in the survey in February 2019, and the survey was closed in approximately 2 weeks once the desired sample of 6000 participants was reached. Minimum sample strata response counts were required for combinations of specialty groups, gender identity, and age. The final sample was deemed representative of practicing US physicians compared with the 2018 American Medical Association’s Physician Masterfile, except for international medical graduates (by 6%). The sampling error was ±1.3% at a 95% confidence level using a point estimate of 50%. The study was approved by the American Institutes for Research Institutional Review Board and deemed exempt from further review because the data were deidentified. This study followed the American Association for Public Opinion Research (AAPOR) reporting guidelines.

The survey allowed physicians to self-disclose their disabilities from a list of 8 possible disability categories using the Americans With Disabilities Act definition. Physicians self-identified race/ethnicity from a list of survey options, and also self-identified their gender assigned at birth as well as their current gender identity. For each category, respondents could select all responses that applied. In addition to demographic characteristics, physicians were asked about their practice characteristics, including employment arrangement, work location (rural or urban), specialty, and use of telehealth services. The 95% CIs were generated for physicians with and without disabilities (2-sided *P* < .05 was considered to be statistically significant). We used Stata SE version 15.1 (StataCorp) to perform unpaired, 2-sided independent *t* tests to compare prevalence rates and means between these groups.

## Results

This study used a representative sample of 6000 physicians, 178 of whom (3.1%; 95% CI, 2.6%-3.5%) self-identified as having a disability. The survey sample included 3768 (62.8%) men, 2055 (34.3%) women, and 20 (0.3%) transgender, genderqueer, or other. The mean (SD) age of participants was 53.0 (12.3) years. The predominant races/ethnicities (not mutually exclusive) were 4148 (69.1%) White, 1347 (22.5%) Asian, and 224 (3.7%) Hispanic, Latino, or of Spanish origin. The disability category most commonly reported was chronic health conditions (54 [30.1%]; 95% CI, 23.3%-36.9%), followed by mobility (51 [28.4%]; 95% CI, 21.7%-35.1%), psychological (25 [14.2%]; 95% CI, 9.0%-19.4%), other disabilities (eg, essential tremors: 24 [13.4%]; 95% CI, 8.3%-18.4%), hearing (22 [12.1%]; 95% CI, 7.3%-17.0%), adult attention-deficit/hyperactivity disorder (19 [10.4%]; 95% CI, 5.9%-14.9%), visual (14 [7.8%]; 95% CI, 3.8%-11.8%), and learning (5 [2.6%]; 95% CI, 0.2%-4.9%). Multiple disabilities (eg, hearing and mobility) were reported by 28 physicians (15.7%; 95% CI, 10.3%-21.1%).

Physicians with disabilities were significantly older than those without disabilities (mean [SD] age, 54.8 [12.7] vs 52.5 [12.2] years) (Table 1). Among physicians with disabilities, 16 (9.2% ) identified as members of a racial or ethnic group underrepresented in medicine, and 26 (14.7%) served on active military duty, either currently or in the past. Ten physicians (5.8%) with disabilities identified as bisexual, and 3 (1.7%) identified as gay or lesbian.

**Table 1.** Demographic Characteristics of Practicing US Physicians With and Without Disabilities, 2019^a^

Characteristic	Physicians with data, No. (%)		*P* value^d^
	With disabilities (n = 178)^b^	Without disabilities (n = 5671)^c^	
Race/ethnicity			
Hispanic, Latino, or Spanish origin	9 (5.1)	155 (2.7)	
American Indian or Alaskan Native	2 (1.0)	7 (0.1)	
Asian	30 (17)	1245 (22)	
Black or African American	6 (3.2)	134 (2.4)	
Native Hawaiian or other Pacific Islander	0	18 (0.3)	
White	115 (64.4)	3832 (67.6)	.38
Multiple races/other	17 (9.4)	269 (4.7)	
Missing	0	12 (0.2)	
Race and ethnicity (aggregated)			
Underrepresented in medicine^e^	16 (9.2)	295 (5.2)	
Gender identity			
Female	59 (33.1)	1996 (35.2)	.92
Male	116 (65.2)	3652 (64.4)	.87
Transgender, genderqueer, or other	3 (1.7)	17 (0.3)	
Missing	0	5 (0.1)	
Sexual orientation			
Bisexual	10 (5.8)	50 (0.9)	
Gay or lesbian	3 (1.7)	108 (1.9)	
Heterosexual or straight	158 (89.7)	5427 (95.7)	.001
Other	5 (2.7)	50 (0.9)	
Missing	1 (0.8)	36 (0.7)	
Age group, y			
≤40	29 (16.1)	1049 (18.5)	
41-50	38 (21.4)	1304 (23.0)	
51-60	35 (19.6)	1336 (23.6)	
61-70	53 (30.0)	1312 (23.1)	
≤70	17 (9.3)	385 (6.8)	
Missing	6 (3.5)	284 (5.0)	
Age, mean (SD), y	54.8 (12.7)	52.5 (12.2)	.009
Military service			
Never served on active duty in the military	151 (84.8)	5259 (92.7)	
Served on active duty in past or currently serving	26 (14.7)	412 (7.3)	<.001
Missing	1 (0.4)	0	

^a^ The source for all data is the analysis of data from the 2019 National Sample Survey of Physicians by one of the authors (Z.N.) (eReference in the Supplement).

^b^ The unweighted count of physicians reporting disability was 169, but after applying the sampling weights (necessary for proper representation of the study population), the weighted count is 178.

^c^ The counts do not sum to 6000 because in 149 cases a value was missing for the disability variables (178 + 5673 + 149 = 6000).

^d^ Calculated from 2-sample *t* tests. However, where no *P* value is listed, *t *tests were not conducted because one of the samples was less than 30.

^e^ Underrepresented race/ethnicity in medicine includes Black; Hispanic, Latino, or Spanish origin; and American Indian or Alaska Native.

Compared with the 5671 physicians without disabilities, higher percentages of the 178 physicians with disabilities reported working in medical schools (14 [8.1%] vs 242 [4.3%]), in nonteaching hospitals (18 [10.0%] vs 366 [6.5%]), and locum tenens (9 [5.0%] vs 76 [1.3%]), but the differences were not statistically significant. Although physicians with disabilities worked fewer hours per week on average (mean [SD], 43.2 [19.9] vs 47.1 [15.5] h/wk; *P* = .001), they had more on-call days (mean [SD], 1.0 [1.5] vs 0.6 [1.2] d/wk; *P* < .001) and were significantly more likely than physicians without disabilities to work in rural areas (31 physicians [17.3%] vs 621 [11.0%]; *P* = .008) (Table 2).

**Table 2.** Practice Characteristics of Practicing US Physicians With and Without Disabilities, 2019^a^

Characteristic	Physicians with data, No. (%)		*P* value^c^
	With disabilities (n = 178)	Without disabilities (n = 5671)^b^	
Academic affiliation	83 (46.8)	2539 (44.8)	.89
Work time excluding calls, mean (SD), h/wk^d^	43.2 (19.9)	47.1 (15.5)	.001
On call, mean (SD), d/wk^e^			
In house	1.0 (1.5)	0.6 (1.2)	<.001
At home	3.4 (2.3)	2.9 (2.4)	.05
Main employment arrangement			
Medical school	14 (8.1)	242 (4.3)	
Teaching hospital	21 (12.0)	763 (13.5)	
Nonteaching hospital	18 (10.0)	366 (6.5)	
Physician group	31 (17.7)	1182 (20.9)	.40
Government	4 (2.4)	150 (2.7)	
Health system	16 (9.2)	742 (13.1)	
Private practice	52 (29.3)	1984 (35.0)	.06
Locum tenens^f^	9 (5.0)	76 (1.3)	
Other	11 (6.4)	162 (2.9)	
Missing	2 (0.04)	0	
Primary work location^g^			
Rural	31 (17.3)	621 (11.0)	.008
Urban	147 (82.7)	5051 (89.1)	
Specialty group			
Primary care specialties	55 (31.2)	1988 (35.1)	.65
Medical specialties	29 (16.3)	934 (16.5)	.84
Surgical specialties	32 (18.2)	1083 (19.1)	.88
Other specialties^h^	62 (34.6)	1667 (29.4)	.38
Use of telehealth services by modality			
Electronic patient communications and consultations	73 (41.0)	2344 (41.3)	.96
Electronic asynchronous consultation, physician to physician	36 (20.1)	594 (10.5)	<.001
Live video visits with patients	18 (10.1)	409 (7.2)	
Live video consultations, physician to physician	7 (3.9)	213 (3.8)	
Remote patient monitoring	10 (5.4)	259 (4.6)	
Other	2 (0.9)	61 (1.1)	
None	71 (39.7)	2863 (50.5)	.004

^a^ The source for all data is the analysis of data from the 2019 National Sample Survey of Physicians by one of us (N.Z.) (eReference in the Supplement).

^b^ The counts do not sum to 6000 because in 149 cases a value was missing for the disability variables (178 + 5673 + 149 = 6000).

^c^ Calculated from 2-sample *t* tests. However, where no *P* value is listed, *t *tests were not conducted because one of the samples was less than 30.

^d^ Work hours excluding calls reflects the hours worked by the respondent in their most recent typical workweek, excluding time spent on call.

^e^ Days on call is the number of days the respondent was on call in their most recent typical workweek.

^f^ Locum tenens refers to a physician working temporarily in another practice.

^g^ US Department of Agriculture Economic Research Service Rural-Urban Commuting Area codes were assigned using the respondent’s main work address.

^h^ Other includes all specialties not in the 3 other categories, such as anesthesiology, emergency medicine, neurology, pathology, physical medicine and rehabilitation, psychiatry, and radiology.

## Discussion

To our knowledge, this is the first study to examine the prevalence of US practicing physicians with disabilities; we estimated an overall disability prevalence of 3.1%. Many of the physicians also identified as members of other groups likely to face discrimination. Given the bias, harassment, and discrimination experienced by groups traditionally underrepresented in medicine,^6^ these findings may have important implications for physicians at the intersection of multiple marginalized identities. Limitations of this study include participant bias and underreporting of disabilities, which may lead to an underestimate of disabilities in the population.

These data provide a benchmark to track growth in this population of physicians. Many physicians with disabilities reported multiple underrepresented identities. Accordingly, research examining harassment, discrimination, and bias is needed to inform efforts to create more inclusive and equitable workplaces and to support retention in the profession.

## Notes

Supplement. **eMethods.** Notes on Sampling Method **eReference.**
