# Error in Funding/Support Statement of Article Information Section

**DOI:** 10.1001/jamanetworkopen.2021.10025

**Published:** 2021-04-15

In the Research Letter titled “Estimated Prevalence of US Physicians With Disabilities,”^1^ published March 12, 2021, information was inadvertently omitted from the Funding/Support statement in the Article Information section. The end of that statement should have parenthetically included the names of the authors to whom the funding was directed: Mrs Nouri, Mr Dill, and Ms Conrad. This article has been corrected.
