# Errors in Results and Tables 1 and 2

**DOI:** 10.1001/jamanetworkopen.2022.32194

**Published:** 2022-08-25

In the Research Letter titled “Estimated Prevalence of US Physicians With Disabilities,”^1^ published March 12, 2021, errors occurred in the Results section and in Tables 1 and 2. In the first sentence of the Results section, the upper 95% confidence limit should have appeared as 3.6% instead of 3.5%. The sentence should have read: “This study used a representative sample of 6000 physicians, 178 of whom (3.1%; 95% CI, 2.6%-3.6%) self-identified as having a disability.” In the last paragraph of the Results section and also in the column headings of Tables 1 and 2, the number of physicians without disabilities was incorrectly given as 5671. The correct number was 5673, as indicated in table footnotes. This article has been corrected.^1^
