# Supplementary material for: Estimated Prevalence of US Physicians With Disabilities
Source: JAMA Netw Open. 2021 Mar 12;4(3):e211254. doi: 10.1001/jamanetworkopen.2021.1254 (PMC7955270; doi:10.1001/jamanetworkopen.2021.1254)
Supplement: Supplement. — eMethods. Notes on Sampling Method eReference. [file jamanetwopen-e211254-s001.pdf]

Supplemental Online Content

Nouri Z, Dill MJ, Conrad SS, Moreland CJ, Meeks LM. Estimated prevalence of US physicians with disabilities. *JAMA Netw Open.* 2021;4(3):e211254.
doi:10.1011/jamanetworkopen.2021.1254

eMethods. Notes on Sampling Method

eReference.

This supplementary material has been provided by the authors to give readers additional information about their work.

© 2021 Nouri Z et al. *JAMA Network Open*.

eMethods. Notes on Sampling Method

The Association of American Medical Colleges (AAMC)'s National Sample Survey of Physicians (NSSP) was conducted for the AAMC by an external firm that recruited active physicians from their own and their partners' proprietary panels of healthcare professionals to obtain a sample of 6,000. Participants received a cash incentive ($25 for primary care physicians; $35 for specialists) for survey completion.

AAMC set a total sample size of 6,000, as well as provided sampling quotas across 24 age-sex-specialty strata based on power calculations using the AMA's Physician Masterfile as a reference (with n=6,000, alpha=0.05, for the two-sided alternative, for paired samples of data, the power is 1.000). A total of 86,951 qualified physicians were invited to participate in the survey in February 2019, and the survey was closed after two weeks once the desired sample of 6000 participants was reached, with minimum sample sizes required by each combination of specialty group, gender and age group. This resulted in a sampling that does not yield a standard response rate because no additional responses were accepted once the strata minimum sample sizes and total sample size were reached. The sampling error for the survey is +/− 1.3% at a 95% confidence level using a point estimate of 50%.

The data were weighted to be representative of all practicing physicians in the U.S. in terms of specialty group, gender, age group and International Medical Graduate (IMG) status using data from American Medical Association's Physician Masterfile 2018. The NSSP was reviewed and approved by the AAMC Institutional Review Board.

eReference.

The National Sample Survey of Physicians, 2019. Association of American Medical Colleges. (All data and rights owned by AAMC.)

© 2021 Nouri Z et al. *JAMA Network Open*.
